# Preferences for Accessing Medical Information in the Digital Age: Health Care Professional Survey

**DOI:** 10.2196/25868

**Published:** 2021-06-19

**Authors:** Evelyn R Hermes-DeSantis, Robert T Hunter, Julie Welch, Roma Bhavsar, Daniel Boulos, Marie-Ange Noue

**Affiliations:** 1 phactMI West Point, PA United States; 2 Ernest Mario School of Pharmacy Rutgers, The State University of New Jersey Piscataway, NJ United States; 3 EMD Serono Inc Rockland, MA United States; 4 Biogen Cambridge, MA United States; 5 Bristol Myers Squibb Lawrenceville, NJ United States; 6 EMD Serono Mississauga, ON Canada

**Keywords:** information-seeking behavior, access to information, internet, physicians, nurses, pharmacists, medical literature, databases, search tools, medical information

## Abstract

**Background:**

Health care professionals (HCPs) routinely have questions concerning the medications they are recommending. There are numerous resources available; however, each has its own advantages and disadvantages.

**Objective:**

The purpose of this survey was to gain knowledge of the preferred methods and sources HCPs use to obtain information concerning medications.

**Methods:**

A total of 511 HCPs (202 physicians, 105 pharmacists, 100 advance practice nurses, 53 registered nurses, and 51 physician assistants) were surveyed through a third-party market research firm. All participants were practicing in the United States. Individuals working for a pharmaceutical company were excluded. The survey collected demographics, frequency of searching medical information, types of questions searched, sources of medical information, and rationale for preferred and nonpreferred sources of medical information. Use of medical information resources were rated on a 5-point ordinal scale. Data were analyzed with descriptive statistics.

**Results:**

Of the 511 respondents, 88.5% (452/511) searched for medical information either daily or several times per week. The most common questions involved dosing and administration, drug-drug interactions, adverse events and safety, clinical practice guidelines, and disease state information. The main rationale for using specific medical websites or apps and general online search engines frequently or very frequently was ease of use (medical websites or apps: 269/356, 75.6%; general online search engines: 248/284, 87.3%). Accuracy was the main rationale for frequent or very frequent use of medical literature search databases (163/245, 66.5%), prescribing labels or information (122/213, 57.3%), and professional literature (120/195, 61.5%). The main reason for rarely or never using specific medical websites or apps and medical literature search databases was unfamiliarity (medical websites or apps: 16/48, 33%; medical literature search databases: 35/78, 45%); for general online search engines, inaccuracy (34/54, 63%); and for prescribing labels or information and professional literature, excessive time (prescribing labels or information : 54/102, 52.9%; professional literature: 66/106, 62.3%). The pharmaceutical company was sometimes used as a resource for medical information. When the medical information department was used, the call center and the website were considered thorough and complete (call center: 14/25, 56%; website: 33/55, 60%). However, the rationale for not using the call center was the time required (199/346, 57.5%) and the website being unfamiliar (129/267, 48.3%).

**Conclusions:**

The driving forces in the selection of resources are accuracy and ease of use. There is an opportunity to increase awareness of all the appropriate resources for HCPs which may aid in their daily clinical decisions. Specifically, pharmaceutical company medical information departments can help fulfill this need by addressing two major challenges with use of the pharmaceutical company: lack of awareness of medical information services and the speed at which responses are disseminated. Overall, there is lack of understanding or appreciation of the range of pathways to obtain published information and knowledge from pharmaceutical company medical information services. Among the many challenges resource champions will face are the ability to effectively make resources and their platforms accessible, known, and useful to the scientific community.

## Introduction

Health care professionals (HCPs) routinely seek medical information concerning the therapies recommended or used to manage and treat their patients. The US Food and Drug Administration (FDA) approved over 100 novel medications between 2019 and 2020 with a trend for increasing annual approvals over the last decade [1]. Meanwhile, medical information resources that HCPs use to address questions and issues in caring for their patients are growing and expanding [2].

HCPs have a wide variety of options for seeking answers to their questions. The number of resources has expanded and now includes drug aggregate platforms (Epocrates, MicroMedex, Up-to-Date, Medscape), medical information departments, professional journals, prescribing labels, electronic health record systems, textbooks, search engine websites, and academic drug information centers [2]. Although the drug aggregate platforms provide a wealth of information, these databases may contain misinformation [3]. In a 2020 study of neurologists, online resources were preferred (96%) compared to offline resources (47%) [4]. According to the 2013 study by Kritz [5], physicians used online resources for medical information; however, access to quality information was a barrier of note.

Medical information services offered by pharmaceutical companies can provide evidence-based, scientifically balanced, accurate, truthful, nonmisleading responses to unsolicited inquiries from HCPs. These unsolicited inquires can be questions concerning the FDA-approved product labels or questions beyond the labeled information. The responses to these inquiries conform with internal procedures and policies as well as with the FDA draft guidance document [6].

Previous literature has suggested that ease of access and quality are important factors when physicians or medical students search for medical information [7]. For example, in a 2009 survey, 92% of physicians reported clicking results toward the top of a page when searching for medical information online [8]. Search engine algorithms are continually updating and adapting in an effort to improve the search results, identify high quality content, and devalue lower-quality content [9]. However, online access alone may not be enough, as barriers such as time constraints confounded by password or account creation requirements on some medical information department websites may impede use of these resources. Another key component noted in past surveys is that HCPs consider current information from a trusted source as high-quality information [7]. Overall awareness, access, and trust have been discussed as important factors HCPs consider when deciding on medical information resources [2]. In a comprehensive literature review of more than 30 studies, Davies et al [10] found that the majority of research focused predominantly on physicians and the type of information they sought. A key barrier to physicians’ search for information was the time needed to perform the search effectively. The studies on this topic [2,7-10], most of which are outdated, highlight the lack of published literature on this subject, particularly evaluations on the search preferences of HCPs apart from those of physicians.

Given the numerous resources HCPs can use today for searching or requesting medical information, there is a need to better understand their search preferences, processes, and barriers to using these resources. Therefore, the purpose of this study was to gain knowledge about the frequency, preferred methods, and most common sources HCPs use to obtain medical information. An additional goal was to evaluate and categorize the rationale of HCP choices with the aim of enhancing medical information services within pharmaceutical companies.

## Methods

A deidentified, web-based, qualitative survey was designed by members of phactMI to collect the opinions of HCPs concerning their search preferences for medical information. Surveys were distributed through a third party platform (Dynata market research organization). Recruitment and participation were communicated via email through Dynata. Participants received a unique identifier that did not reveal their identity to the study team. The survey was administered once and was open for 1 week in March 2019.

HCPs (physicians, pharmacists, nurse practitioners or advance practice nurses, registered nurses, and physician assistants) received an email inviting them to participate in the survey. These HCPs were registered with the third-party surveying platform, Dynata. The survey was distributed to a panel of verified HCPs and was available until the prespecified convenience sample was obtained. A sample size of 202 physicians, 105 pharmacists, 100 advance practice nurses, 53 registered nurses, and 51 physician assistants was chosen and felt to be representative of those HCPs who contact the typical mid- and large-size pharmaceutical companies’ medical information departments. Registered HCPs responded to qualifying questions based on the inclusion and exclusion criteria. Participants were included based on their profession (practicing physician, pharmacist, nurse practitioner or advanced practice nurse, registered nurse, and physician assistant) and their country of practice (the United States). Participants who were not currently practicing or who were employed by a pharmaceutical or biopharmaceutical company were excluded. Once 511 qualified respondents completed the survey, the recruitment period ended. Participants were asked for their consent to participate in the survey prior to survey administration. Dynata follows the International Chamber of Commerce/European Society for Opinion and Marketing Research (ICC/ESOMAR) International Code on Market, Opinion, and Social Research and Data Analytics.

The survey collected information regarding HCPs’ demographics (HCP type, years of practice, specialty, and practice setting) and their search preferences and processes. The survey took about 10 minutes and consisted of 13 questions. Questions in the survey included frequency of searching for medical information, inquiry categories, sources of medical information used, and rationale for preferred and nonpreferred sources of medical information. The survey consisted of multiple choice questions, ranking, and, to a lesser extent, a free-text field. Based on responses given throughout the survey, additional information was ascertained regarding reasons why or why not certain sources of medical information were preferred or not preferred. See Multimedia Appendix 1 for the full list of survey questions and available responses.

Descriptive analysis of data was conducted. Data were analyzed based on the entire cohort as well as on profession, specialty, and years in practice. Chi-square and Fisher exact tests were used for categorical data.

## Results

### Demographics

Based on the convenience sample set, a total of 511 health care professionals in the United States were included in the survey, comprising physicians (202/511, 39.5%), pharmacists (105/511, 20.5%), nurse practitioners or advanced practice nurses (100/511, 19.6%), registered nurses (53/511, 10.4%), and physician assistants (51/511, 10.0%). The most common practice settings represented included private practice (211/511, 41.3%), community hospitals (127/511, 24.9%), and academic or teaching hospitals (102/511, 20.0%). Consistent with the dominant practice settings in this survey, almost half (249/511, 48.7%) of all respondents worked in primary care. Moreover, 80.2% (410/511) of those surveyed had been in practice for ≥ 11 years. See Table 1 for additional details.

**Table 1:** Demographics.

Practice setting^a^			All HCPs^b^, n (%)		Physicians, n (%)		Pharmacists, n (%)		NP/APN^c^, n (%)		RN^d^, n (%)		PA^e^, n (%)
All settings			511 (100)		202 (39)		105 (21)		100 (20)		53 (10)		51 (10)
Private practice			211 (41)		132 (65)		1 (1)		41 (41)		8 (15)		29 (57)
Academic/teaching hospital			102 (20)		40 (20)		16 (15)		24 (24)		13 (25)		9 (18)
Community hospital			127 (25)		60 (30)		21 (20)		21 (21)		17 (32)		8 (16)
Other^f^			204 (40)		33 (16)		84 (80)		26 (26)		19 (36)		14 (27)
**Specialty practice**													
	Primary care	249 (49)		102 (50)		50 (48)		57 (57)		12 (23)		28 (55)	
	Oncology/hematology	36 (7)		10 (5)		13 (12)		4 (4)		7 (13)		2 (4)	
	Cardiology	25 (5)		11 (5)		0 (0)		7 (7)		5 (9)		2 (4)	
	Psychiatry	17 (3)		11 (5)		2 (2)		3 (3)		1 (2)		0 (0)	
	Orthopedics	10 (2)		5 (2)		0 (0)		2 (2)		0 (0)		3 (6)	
	General surgery	10 (2)		6 (3)		1 (1)		0 (0)		3 (6)		0 (0)	
	Endocrinology	9 (2)		3 (1)		1 (1)		3 (3)		1 (2)		1 (2)	
	Pulmonology	5 (1)		5 (2)		0 (0)		0 (0)		0 (0)		0 (0)	
	Neurology	5 (1)		4 (2)		0 (0)		1 (1)		0 (0)		0 (0)	
	Rheumatology	4 (1)		7 (3)		1 (1)		0 (0)		0 (0)		1 (2)	
	Other	141 (28)		43 (21)		37 (35)		23 (23)		24 (45)		14 (27)	
**Years in practice**													
	HCPs >20 years	230 (45)		90 (45)		62 (59)		35 (35)		30 (57)		13 (25)	
	HCPs 11-20 years	180 (35)		68 (34)		32 (30)		40 (40)		14 (26)		26 (51)	
	HCPs <11 years	101 (20)		44 (22)		11 (10)		22 (22)		8 (15)		12 (23)	

^a^More than one practice setting could be selected.

^b^HCPs: health care professionals.

^c^NP/APN: nurse practitioner/advanced practice nurse.

^d^RN: registered nurse.

^e^PA: physician assistant.

^f^Other practice settings included health maintenance organization (n=73), pharmacy hospital (n=23), pharmacy retail (n=64), managed care (n=6), research (n=4), long-term care (n=22), and other (n=12).

### Frequency of Search

**Figure 1:**
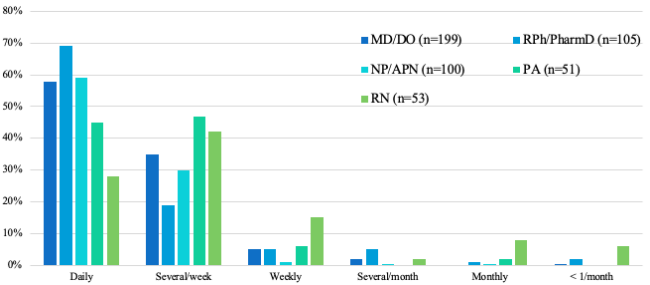
Frequency of medical information searches. APN: advanced practice nurse; DO: Doctor of Osteopathic Medicine; MD: medical doctor; NP: nurse practitioner; PA: physician assistant; PharmD: Doctor of Pharmacy; RN: registered nurse; RPh: registered pharmacist.

Of the 511 respondents, 452 (88.5%) searched for medical information either daily or several times per week (Figure 1). In particular, 92.6% (187/202) of physicians surveyed search for medical information either daily or several times per week, compared to 87.6% (92/105) of pharmacists, 89.0% (89/100) of advance practice nurses, 70% (37/53) of registered nurses, and 92% (47/51) of physician assistants (*X*^2^_4_=49.51; *P*<.001). Overall, 90.1% (91/101) of respondents with 10 years of practice or less and 88.0% (361/410) of respondents with 11 or more years of practice searched for medical information daily or several times per week.

### Inquiry Type

**Figure 2:**
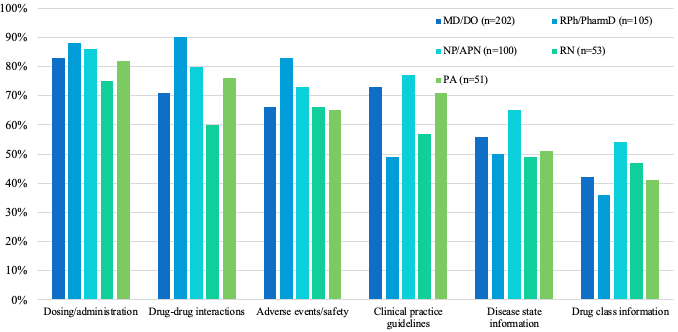
Information typically sought by healthcare professionals. APN: advanced practice nurse; DO: Doctor of Osteopathic Medicine; MD: medical doctor; NP: nurse practitioner; PA: physician assistant; PharmD: Doctor of Pharmacy; RN: registered nurse; RPh: registered pharmacist.

The most common questions across all HCP types concerned dosing or administration (428/511, 83.8%), drug-drug interactions (389/511, 76.1%), adverse events and safety (361/511, 70.6%), clinical practice guidelines (342/511, 66.9%), and disease state information (283/511, 55.4%; Figure 2). Overall, there were no significant differences between the professions and the type of information they were looking for.

#### Search Tools

**Figure 3:**
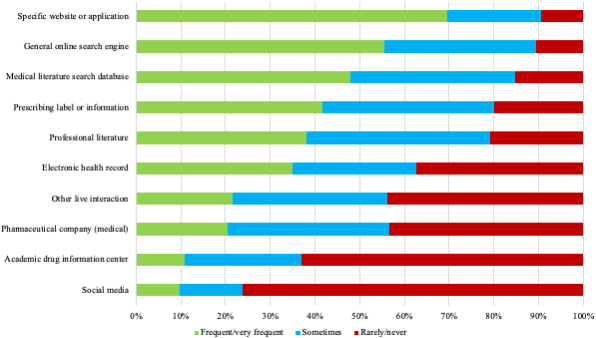
Frequency of use of major information sources.

More than half (58.7%, 300/511) of respondents indicated searching for medical information from a desktop, laptop, or workstation a majority (ie, >50%) of the time as opposed to the 25.6% (131/511) of respondents who used a mobile device the majority of the time. Specific medication websites or apps were frequently or very frequently searched by 69.7% (356/511) of the respondents. Furthermore, 55.6% (284/511) of HCPs surveyed use general online search engines such as Google or Yahoo frequently or very frequently. Medical literature search databases, prescribing labels or information, professional literature, and company resources were accessed less frequently (Figure 3).

**Figure 4:**
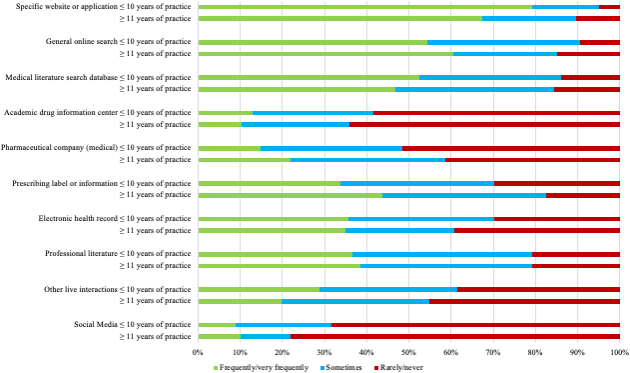
Search option preference based on years of practice.

Differences in the search option used were observed based on the number of years in practice. Overall, 24.8% (25/101) of the less experienced respondents (10 years of practice or less) versus 36.8% (151/410) of the respondents with 11 years or more of practice reported sometimes using general online search engines; 79.2% (80/101) of the less experienced respondents versus 67.3% (276/410) of the more experienced respondents reported frequent or very frequent use of specific websites or app for their searching needs; 29.7% (30/101) of the less experienced respondents versus 39.3% (161/410) of the more experienced respondents reported rarely or never using electronic health record information; and 67.3% percent (69/101) of the less experienced respondents reported rarely or never using social media versus 78.0% (320/410) of the more experienced group (Figure 4). Differences in the search option used stratified by the number of years in practice did not reach statistical significance.

**Figure 5:**
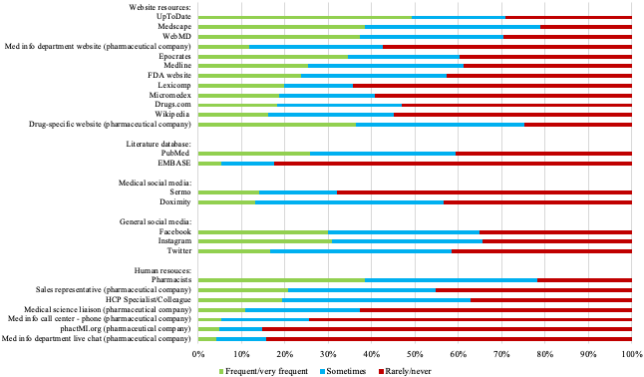
Frequency of use of source information. FDA: Food and Drug Administration; HCP: health care professional.

In the analysis regarding the frequency of use of search options, UpToDate ranked highest among all individual sources of medical information (excluding general online search engines). The drug-specific website was the most frequently used resource by those contacting the pharmaceutical company directly. PubMed and MEDLINE were the most popular literature search databases used. Facebook, Sermo, and Instagram were the most popular social media platforms used (Figure 5).

#### Reasons for Frequent or Infrequent Use of Search Options

Ease of use was the rationale for the frequent or very frequent use of specific medication websites or apps (269/356, 75.6%) and general online search engines (248/284, 87.3%; Table 2). Accuracy was the major rationale for frequent or very frequent use of medical literature search databases (163/245, 66.5%), prescribing labels or information (122/213, 57.2%), and professional literature (120/195, 61.5%). The main reason for infrequent (rarely or never) use of specific medication websites or apps (16/48, 33%) and medical literature search databases (35/78, 44.9%) was unfamiliarity; for general online search engines, inaccuracy (34/54, 63%); and for prescribing labels or information (54/102, 52.9%) and professional literature, time (66/106, 62.2%; Table 2).

In general, there was low usage of the pharmaceutical company (medical information website, medical science liaison, or sales representative). HCPs in the setting of a hospital pharmacy (6/23, 26%) and retail pharmacy (16/64, 25%) were more likely than those in an academic hospital (16/102, 15.6%) to use the services of the pharmaceutical company frequently or very frequently. When the medical information department was used, the medical information department call center and the website were considered thorough or complete (14/25, 56% and 33/55, 60%); however, the barriers included excessive time (199/346, 57.5%; 79/267, 29.6%) or unfamiliarity with the service (118/346, 34.1%; 129/267, 48.3%; Table 2). Similar findings were reported regarding the pharmaceutical company field medical teams. For HCPs who frequently or very frequently contact a sales representative when searching for medical information, accessibility of the sales representative was the most stated rationale provided.

There was generally low usage of the medical information department live chat service, with only 3.7% (19/511) of HCPs reporting that they use this service frequently or very frequently. When used, accessibility was the main driver (13/19, 68%). For the majority of respondents who indicated having rarely or never contacted the pharmaceutical company via live chat, 33.8% (133/393) were unfamiliar with the service and 51.9% (204/393) noted it took too long.

Free-text entries were reviewed. Additional information regarding practice settings have been included under demographics (see Table 1); however, data obtained from other text fields did not provide any useful information and have not been summarized due to the limited range and number of entries.

**Table 2:** Rationale for frequent or very frequent or rarely/never use (N=511).

Rationale by use frequency			General online search, n (%)		Medical literature database, n (%)		Specific web app, n (%)		Med info^a^ call center, n (%)		Med info^a^ website, n (%)		Medical science liaison, n (%)		Drug website, n (%)
**Frequent/very frequent^b^**			284 (56)		245 (48)		356 (70)		25 (5)		55 (11)		50 (10)		169 (33)
	No other option	9 (3)		3 (1)		6 (2)		0 (0)		3 (5)		1 (2)		8 (5)	
	Familiarity	148 (52)		104 (42)		210 (59)		7 (28)		12 (22)		12 (24)		43 (25)	
	Responsive/quick	177 (62)		57 (23)		154 (43)		11 (44)		16 (29)		19 (38)		58 (34)	
	Ease of use	248 (87)		100 (41)		269 (76)		9 (36)		24 (44)		19 (38)		92 (54)	
	Accuracy	53 (19)		163 (67)		226 (63)		11 (44)		27 (49)		24 (48)		70 (41)	
	Thorough/complete	58 (20)		137 (56)		197 (55)		14 (56)		33 (60)		27 (54)		67 (40)	
	Accessibility	168 (59)		97 (40)		211 (59)		13 (52)		25 (45)		27 (54)		94 (56)	
**Rarely/never^b^**			54 (11)		78 (15)		48 (9)		346 (68)		267 (52)		290 (57)		115 (23)
	Inaccurate	34 (63)		1 (1)		5 (10)		7 (2)		3 (1)		7 (2)		9 (8)	
	Not available at my organization	5 (9)		18 (23)		8 (17)		16 (5)		30 (11)		56 (19)		8 (7)	
	Not thorough enough	28 (52)		3 (4)		6 (13)		23 (7)		19 (7)		18 (6)		34 (30)	
	Difficult to use	3 (6)		21 (27)		3 (6)		57 (16)		37 (14)		26 (9)		12 (10)	
	Difficult to access	3 (6)		22 (28)		12 (25)		89 (26)		60 (22)		67 (23)		18 (16)	
	Unfamiliar with method	3 (6)		35 (45)		16 (33)		118 (34)		129 (48)		120 (41)		30 (26)	
	Takes too long	15 (28)		32 (41)		10 (21)		199 (58)		79 (30)		90 (31)		31 (27)	

^a^Med info: medical information.

^b^Percentages in this row are derived from the total number of responses (N=511).

## Discussion

### Principal Findings

With the lack of recently published information on search preferences for HCPs, this study provides insights into frequency, preferred methods, and commonly used sources that a broad range of HCPs used to obtain medical information. Overall, 88.5% (452/511) of all HCPs, including almost 95% of physicians, search for medical information either daily or several times per week. Data from this study show that HCPs are using only a few of the aforementioned resources, and many valuable assets are being underused. Medical literature search databases, prescribing labels or information, professional literature, and pharmaceutical company resources were used less frequently compared to other resources, such as general online search engines or specific websites and apps. It is important to note here that general search engines may be used by HCPs to access specific web-based resources such as Medscape and WebMD. Potential reasons for low usage can vary from access issues with literature databases, inability to find a specific answer to a question from the label, or a lack of awareness of pharmaceutical resources such as medical information services or access to company representatives. With the rapidly expanding bank of scientific data, it is becoming increasingly important to have access to information from credible sources.

Taking a closer look at the use of the pharmaceutical company as a resource, less frequent use was due in part to perceived barriers which include the length of time it takes to produce data in response to a specific unsolicited question and the unfamiliarity of the HCPs with the medical information service provided. Previous research has documented that HCPs who use medical information services identify the information as trustworthy [11,12]. However, among the HCPs who had not used medical information services, the top 3 reasons were bias (55%), lack of awareness (41%), and lack of transparency (30%) [12]. The technological advancement and rise in new tools in the pharmaceutical and biotech industry have the potential to better address HCPs’ needs, provided that some work is done to resolve the existing barriers.

### Limitations

One limitation to this study is the inclusion of only US HCPs. In addition, the demographics of the various HCPs, including physicians (202/511, 39.5%), pharmacists (105/511, 20.5%), nurse practitioners or advanced practice nurses (100/511, 19.6%), registered nurses (53/511, 10.4%), and physician assistants (51/511, 10.0%), was not equally distributed and might have skewed the data towards physician preferences. Another limitation is that the survey was qualitative in nature; therefore, some of the terminology used within the questions could have been subject to personal interpretation. For example, the definition of “ease of use” might have been influenced by an individual’s subjective perception. Another point to consider is that no information on the current level of digital tool usage was gathered from the respondents, so it is not clear if the results might have been influenced by a low level of familiarity with digital tools in general. Additional insights from a larger-scale study with HCPs worldwide would be of interest for comparison.

It is also important to realize the survey was conducted in March 2019, prior to the current global COVID-19 pandemic. Current remote workflow and reliance on technology could alter the responses of HCPs if the survey were repeated today, and it is unclear what the long-term consequences and duration of these changes will be.

### Conclusions

Research has shown that accuracy and ease of use are the driving forces for HCPs in choosing resources for daily use. Data from this study show an opportunity to increase awareness of all the appropriate resources tailored to HCPs which may aid in their daily clinical decisions. In addition, there is an opportunity for medical information departments from pharmaceutical and biotech companies to help fulfill this need by addressing two major challenges in the use of pharmaceutical companies as a resource: lack of awareness of these medical information services and the speed at which responses are disseminated to HCPs. There is a potential opportunity for medical information departments and field medical teams (ie, medical science liaisons) to work together to overcome these perceived barriers through bringing awareness to the service and increasing accessibility to HCPs by emphasizing the point that medical teams are to only provide tailored scientific data in a nonpromotional manner that most suits their work style and demand [6].

With a significant push towards evidence-based medicine, there remains a need for a unified source for medical information that meets the shifting workstyle needs of practitioners. Optimal treatment decisions are incumbent on current, high-quality, nonpromotional data. The results of this research identified gaps in understanding the numerous ways HCPs obtain published data and the limited knowledge of medical information services provided by pharmaceutical and biotech companies. Among the many challenges resource champions will face are the ability to effectively make resources and their platforms accessible, known, and useful for the scientific community. This will further shape and impact the future of patient outcomes.

## Appendix

Multimedia Appendix 1 Survey questions and answer options.

## Abbreviations

HCP: health care professional
ICC/ESOMAR: the International Chamber of Commerce/European Society for Opinion and Marketing Research
