# Supplementary material for: Preferences for Accessing Medical Information in the Digital Age: Health Care Professional Survey
Source: J Med Internet Res. 2021 Jun 19;23(6):e25868. doi: 10.2196/25868 (PMC8386376; doi:10.2196/25868)
Supplement: Multimedia Appendix 1 [file jmir_v23i6e25868_app1.doc]

Appendix 1: Survey Questions and Answer Options

| Q1 | How often do you search for medical information (including, for example, online search, live interactions, print resources, etc.)?   1. Daily 2. Several times per week 3. Weekly 4. Several times per month 5. Monthly 6. Less than once per month |
| --- | --- |
| Q2 | When you search for medical information, what type of information do you typically look for?  (check all that apply)   1. Dosing/Administration 2. Drug stability 3. Pharmacokinetics/Pharmacodynamics/Mechanism of action 4. Adverse events/Safety 5. Efficacy 6. Drug-drug interactions 7. Use in special populations (including comorbid conditions) 8. Drug class information 9. Disease state information 10. Clinical practice guidelines 11. Clinical trial information 12. Other (please specify) |
| Q3 | Please provide additional details about the type(s) of medical information you typically search for. What types of questions are you looking to answer? *Free Text* |
| Q4 | When searching for medical information online, what percentage of the time do you use the following?  (Enter and sum to equal 100%)   1. Mobile device 2. Desktop/laptop/workstation |
| Q5 | When you search for medical information, how often do you use each of the following options?  Likert scale: 1 (Never), 2 (Rarely), 3 (Sometimes), 4 (Frequently), 5 (Very frequently)   1. General online search engine (ie Google, Yahoo) 2. Medical Literature Search Database (PubMed, etc.) 3. Specific Website or Application (Epocrates, Wikipedia, Micromedex, Lexicon, UptoDate, phactMI.org, etc.) 4. Prescribing label/information 5. Pharmaceutical Company (Med Info website, MSL, Sales representative) 6. Other live interaction (eg consult with pharmacist, colleague, specialist) 7. Academic drug information center 8. EHR (electronic health record) 9. Professional literature (ie journals) 10. Other (specify) – free text |
| Q6 | When using a medical literature search database, how often do you use each of the following options? Likert scale: 1 (Never), 2 (Rarely), 3 (Sometimes), 4 (Frequently), 5 (Very frequently) or “This is not available to me”   1. PubMed 2. EMBASE 3. MedLine 4. Other (specify) |
| Q7 | When using a specific website or application, how often do you use each of the following options? Likert scale: 1 (Never), 2 (Rarely), 3 (Sometimes), 4 (Frequently), 5 (Very frequently) or “This is not available to me”   1. Epocrates 2. UpToDate 3. Micromedex 4. Wikipedia 5. Medscape 6. WebMD 7. Lexicomp 8. Drugs.com 9. FDA Website 10. phactmi.org website 11. Other (specify) |
| Q8 | When obtaining medical information from a Pharmaceutical Company, which do you use?   1. Medical Information Department Call Center via telephone (e.g., 1-800 number) 2. Live chat (text or video) with Medical Information Department Call Center 3. Medical Information Department Website 4. Medical Science Liaison (MSL) or similar clinical/medical field member 5. Sales Representative 6. Drug-Specific Website (eg drugname.com) 7. Other (specify) |
| Q9 | When using other live interactions as a source of medical information, how often do you use each of the following options? Likert scale: 1 (Never), 2 (Rarely), 3 (Sometimes), 4 (Frequently), 5 (Very frequently) or “This is not available to me”   1. HCP Specialist/colleague 2. Pharmacist 3. Other (please specify): ­­­­_____________________ |
| Q10 | When obtaining medical information from an EHR/EMR, which do you use?   1. Epic 2. Cerner 3. Other (please specify) _____________________ |
| Q11 | When using social media, how often do you use each of the following options? Likert scale: 1 (Never), 2 (Rarely), 3 (Sometimes), 4 (Frequently), 5 (Very frequently)  a. Facebook b. Twitter c. Sermo d. Doximity e. Instagram |
| Q12 | You stated previously that when searching for medical information you use ________ frequently or very frequently. Why do you prefer to get your medical information from _____?   1. Accuracy 2. Thorough/complete 3. Ease of use 4. Accessibility 5. Responsive/quick 6. Familiarity 7. Because there is no other option (lack of access to other resources) 8. Other (please specify): _____________________ |
| Q13 | You stated previously that when searching for medical information you use _____ rarely or never. Why do you not use _____ to search for medical information?   1. Inaccurate 2. Not thorough enough 3. Difficult to use 4. Difficult to access 5. Takes too long 6. Unfamiliar with this method 7. This method of searching is not available at my organization 8. Other (please specify): _____________________ |
